# Clinicians’ views of factors influencing decision-making for CS for first-time mothers—A qualitative descriptive study

**DOI:** 10.1371/journal.pone.0279403

**Published:** 2022-12-28

**Authors:** Sunita Panda, Cecily Begley, Deirdre Daly

**Affiliations:** School of Nursing and Midwifery, Trinity College Dublin, Dublin, Ireland; ESIC Medical College & PGIMSR, INDIA

## Abstract

Clinicians’ perspectives of the reasons for performing caesarean section (CS) are fundamental to deepening knowledge and understanding of factors influencing decision-making for CS. The aim of this study was to explore midwives’ and obstetricians’ views of factors influencing decision-making for CS for first-time mothers. A qualitative descriptive study with semi-structured one-to-one audio-recorded interviews was used to gather data from clinicians (15 midwives and 20 senior obstetricians). Following research ethics committee approval, clinicians, who were directly involved in the decision-making process for CS during the period of data collection, were purposively selected from three maternity units in the Republic of Ireland between June 2016 to July 2017. The interviews were transcribed verbatim and analysed thematically. Three interrelated themes with several subthemes reflective of clinicians’ views and experiences emerged following data analysis. These were: ‘A fear factor’ describing clinicians’ fear of adverse outcomes and subsequent litigation, ‘Personal preferences versus a threshold–clinician driven factors emphasising the influence of clinicians’ personal beliefs, and ‘Standardised versus individualised care–a system perspective’ explaining the effects of, or lack of, organisational policy and its direct and indirect impact on the decision-making process. Findings show that decisions to perform a CS are, on occasion, based on clinicians’ personal beliefs and interpretation, similar to findings from other published literature. Consideration of broader issues related to organisational, socio-cultural and political context is essential when seeking solutions to the rising CS rates. The findings will enable clinicians to reflect on their day-to-day practice, in order to look for modifiable factors that influence their decision-making, and help women understand the multitude of factors that can lead to a decision to perform a CS. Findings will also contribute to the development of the ‘next step action’ and assist in devising future intervention studies to reduce any unnecessary CSs.

## Introduction

Optimising women’s childbirth experiences while ensuring safe outcomes for mothers and babies is a primary goal in maternity services [1, 2]. However, over the last few decades, more and more women are giving birth to their first baby by CS with lack of clarity around the factors influencing decision-making [3] and lack of evidence of additional benefits to mothers and babies [4, 5]. Research on birth by CS has revealed an increase in both the type and severity of postpartum morbidity, compared to women who birth vaginally [6, 7], complexities around decision-making for future births [8], increased rehospitalisation [9, 10], and increased healthcare costs [11, 12], yet the rising trend has become inevitable in most countries [13]. With the growing concerns around the rising trend of CSs, particularly for first-time mothers, and a lack of clarity around the factors involved in the process of arriving at the decision, it is crucial to get a deep insight into the decision-makers’ perspective. This is essential in order to identify the factors that can help reduce unnecessary first-time CSs safely and will subsequently lead to a reduction in repeat CSs. The concepts ‘Too little, too late (TLTL)’ and ‘Too much, too soon (TMTS)’, introduced by Miller *et al*. (2016) [14] describe the underuse of CS (TLTL) in some parts of the world with associated harm to mothers and babies, and overuse of CS (TMTS) in other parts with increased morbidities for women and newborns. Addressing the issues around these two concepts (TLTL and TMTS) is essential to optimise the appropriate use of CS. Decision-making for CS is influenced by a number of poorly understood complex factors [15], with limited evidence around factors that influence decision-making for first-time mothers [16], which emphasises the need to acknowledge that understanding obstetricians’ and midwives’ concerns are fundamental and essential to reducing unnecessary CSs [3, 14, 17, 18]. Therefore, this study aimed to explore the factors that influenced decision-making for CS for first-time mothers from the perspectives of clinicians (midwives and obstetricians), the key decision-makers.

## Materials and methods

A descriptive qualitative design was used, which consisted of one-to-one in-depth interviews with clinicians (obstetricians and midwives) who were involved in the decision-making process for CS in the selected study sites. This approach was chosen to describe and explore the ‘*what*’, ‘*why*’, *‘how’* and ‘*where*’ of the phenomenon of interest, ‘*factors influencing decision-making for CS*’ [19].

### Settings and participants

The study settings were two large (approximately 8,500 births per annum) and one medium (approximately 3000 births per annum) sized maternity hospitals in the Republic of Ireland. The population in these settings included women from urban and rural areas, with both high and low obstetric risks. The study sample included clinicians (midwives and obstetricians) purposively selected from the three study settings based on the following criteria; labour ward midwives with all levels of experience and obstetricians (consultant obstetricians and senior registrars) who were involved in the decision-making process for CS. Midwives who were not practising in the labour ward at the time of data collection and doctors who were employed as Senior House Officers or who were not involved in clinical decision-making for CS at the time of data collection were excluded from the study.

Midwives are the primary care providers for all women during labour and birth regardless of the type of care (Public, semi-private or private) and level of complexity (low-risk or high-risk pregnancies). There are three maternity care packages available (public, semi-private and private maternity care at two sites and public and private maternity care at one site). Public care is free to all women who are residents in the Republic of Ireland, and women in public care are cared for by a team of midwives and obstetricians. The consultant obstetrician is responsible for decision-making for women who attend for care privately. Midwives’ role includes promoting normality, optimising the woman’s experience of childbirth, decision-making with all women with low-risk profile, seeking review by obstetricians when deviations from normal physiological process are suspected or detected, and working with the obstetric team to facilitate safe outcome for mother and baby. They are thus involved in decision-making for all women with medium and high-risk profiles but the obstetrician would take the lead in decisions.

### Recruitment and data collection

Following Research Ethics Committee approval from the university and the three study settings, the study information (invitation, study information and willingness to participate form) was provided to all eligible midwives and obstetricians. The Director of Midwifery/Nursing had agreed to act as gatekeeper to the midwives, and the Master/Senior Obstetrician had agreed to act as gatekeeper to obstetricians. The study information (S1 Appendix) contained a clear outline of the purpose of the study, what was involved and participants’ rights to decline to take part in or withdraw from the study, at any point. Clinicians interested in taking part were asked to return a completed consent form or ‘Willingness to participate form’ (S2 Appendix), or to send the researcher a text message. The researcher acknowledged receipt of completed consent/willingness to participate forms or text messages and arranged a convenient interview date, time and venue. Before the interview started clinicians were given the opportunity to ask questions, and the purpose of the interview was clarified prior to commencing each interview. Written consent was obtained prior to the interview, and interviews continued until no new major themes emerged.

Previous experience of being a labour and birth suite midwife and of conducting interviews with clinicians, and formal training on conducting qualitative interviews and analysing interview data helped the researcher to conduct in-depth interviews and analyse the data to meet the aim of this research. This was further facilitated by regular discussions and guidance from the co-authors (DD and CB, both academic midwives with extensive clinical experience) through debriefing and maintaining an audit trail of decisions made through data collection process. Interviews were conducted over a 12 month period between July 2016 to June 2017 using an interview guide (S3 Appendix) developed from the literature. One interview guide was used for interviewing both, midwives and obstetricians, because the focus was on decision-making. Although the scopes and practice of midwives and obstetricians are different, both worked within teams and do not make decisions in isolation. The use of one interview guide facilitated consistency in exploring clinicians’ views and comparison of similarities and discrepancies in views of midwives and obstetricians. The interview guide included open-ended questions such as ‘*Tell me about your role in decision-making for CS in nulliparous women*?’ and probing questions such as *‘Can you tell me more about that*?*’* or *‘Can you explain that to me in a little more detail*?’ were used to facilitate discussion. Terms such as ‘*fear of litigation*’ or ‘*hospital guidelines or policy’* were also used as prompts, when appropriate, to facilitate the flow of discussion. Recruitment to the study ceased when data saturation was reached.

### Decision trail

A reflective diary was maintained following each interview to guide the later interviews. Rigour was assured through maintaining an audit trail of decisions made, with peer debriefing with the co-authors, clinicians, and other qualitative research experts.

### Ethical approval and data protection

Ethical approval to conduct the study was granted by the lead author’s university (Ref:140905) and by the participating hospitals’ (Ref:36–2014, 900, 2015–010). Participants’ anonymity was maintained by assigning interview codes (*ID01*, *ID02*, etc.). Interview recordings were transcribed using a professional transcriber who had signed a confidentiality agreement with the university in advance. All data (audio recordings and interview transcripts) were stored securely in accordance with the Irish Data Protection Act 2018 (http://www.irishstatutebook.ie/eli/2018/act/7/enacted/en/html) and managed according to the General Data Protection Regulation (GDPR) Act, 2018. (https://gdpr-info.eu/). NVivo© software package was used to manage interview data.

### Data analysis

Transcripts were read and re-read against the audio recordings to ensure accuracy. Transcripts were coded, categorised (S4 Appendix) and analysed thematically to explore clinicians’ views of factors that influenced their decision to perform a CS in nulliparous women.

### Rigour and trustworthiness

A rigorous and trustworthy thematic analysis is a process of interpreting and representing textual data [20]. Three authors independently coded four randomly selected transcripts and discussed and agreed to the preliminary themes to ensure confirmability and that there were sufficient justifications to arrive at the findings from the interview data which informed analysis of the remaining data. Following analysis of all the interview data, the authors discussed and agreed to the final themes. For member checking clinicians were sent a synopsis of the key findings along with a purposefully designed feedback form to assess their views on the themes and subthemes derived and asked to comment on the extent to which their views were reflected in the key findings. Most clinicians provided positive feedback on the findings and described that the findings represented and interpreted their views very well. The results of member checking were incorporated into the final findings. This was done to ensure transparency and authenticity by acknowledging views of the clinicians who responded to the member checking process.

## Results

### Clinicians’ characteristics

Individual in-depth interviews were conducted with 35 clinicians (20 obstetricians and 15 midwives) recruited from the three study sites. Thirty-three clinicians were interviewed by telephone and two clinicians took part in face-to-face interviews, as preferred. The length of the interviews ranged from 1 hour 27 minutes to 37 minutes, with an average duration of 62 minutes. A majority of the obstetricians (n = 13/20, 65%) and midwives (n = 12/15, 80%) had over five years’ experience at the time of data collection (S1 Table).

### Thematic analysis

Three interrelated key themes representing clinicians’ views of factors influencing decision-making for CS were derived. These were ‘A fear factor’; ‘Personal preferences versus a threshold—clinician driven factors’; ‘Standardised versus individualised care–a system perspective’. Each of these themes had several subthemes (S2 Table).

#### Theme 1 A fear factor

A perceived fear of adverse outcomes and/or legal implications, influenced by clinicians’ past experience, society and media, were reported to be a major influencing factor contributing to the decision-making for CS. Three subthemes; ‘Fear of litigation and/or adverse outcome’; ‘Ever present—influence of past experience’; and ‘Influence of media—personal and professional consequences’ were identified.

*Subtheme 1*.*1 Fear of litigation and/or adverse outcome*. Fear of adverse outcome from vaginal births, and possible legal consequences/litigation, were reported by all the clinicians to be major influencing factors on how they practised and/or in their decision-making to perform a CS. The two causes of fear appeared to be very closely linked in the minds of clinicians.

“*Fear of litigation is huge now*… *So you know you do have to practise defensively sometimes…it’s better to do a caesarean that’s not necessary than…you end up with cerebral palsy or something awful like that*.*” (Midwife 11*)

“*I certainly think the threshold for you know*, *allowing certain things to kind of come to a more natural conclusion has changed because of people’s fear of…the legal implications*.*” (Senior Obs Reg 7)*

“*I think our society*, *we have become more litigious…nobody wants to stand in the court and defend themselves*. *So definitely one of the reasons why the rate of caesarean section is going over the board is the fear of litigation*.*” (Senior Obs Reg 6)*

Although a few midwives viewed ‘fear’ as a learned behaviour from their senior colleagues and working environment, mostly litigation was viewed as an inevitable part of defensive practice; however, a few clinicians felt it did not influence their decision-making to perform a CS.

“*I feel that the fear of litigation has been brought upon me by the senior staff*. *The fear has been embedded in me by them…I think that’s a learned behaviour*. *I don’t think you go into midwifery fearing your job*.*” (Midwife 13)*

“*I think that those who are working in obstetrics…appreciate the fact that…we will be subject to litigation*, *no matter what we do*. *And it’s kind of like part of what you live with*. *It’s part of the job*.*” (Consultant Obstetrician 16)*

“*Potential for legal action is there whether you do*, *or…don’t do a caesarean*. *So*, *I don’t think it influences your decision*…*You can equally have a disastrous caesarean in labour*…*So I don’t really let that influence my decision about caesareans or not*.*” (Consultant Obstetrician 10)*

There was a mixed opinion about the influence of fear of litigation, and variations in practice and decision-making, among seniors versus junior clinicians.

“*When they* [midwives] *get more experience…they…get a bit more fearful*. *So*, *I’d say more senior staff would be a bit more worried about litigation*.*” (Mid 10)*

“*Very…senior consultants would be less inclined to section women straight away without a hard indication*, *whereas younger consultants would be sectioning women for softer indications*.*” (Senior Obs Reg 6)*

In general, although there were variations in practice, most clinicians described practising defensively to avoid adverse outcomes and potential legal consequences.

*Subtheme 1*.*2 Ever present—influence of past experience*. Clinicians’ past experience of an adverse outcome remained with them forever and influenced their decision-making, often changing their practice for the rest of their professional life.

“*It* [fear] *comes with certain experiences*. *If someone has an experience of a case being taken* [to the court of law]*…that will have a huge impact on them*.*” (Mid 14)*

“*If you have just had a bad outcome a week or two ago*, *you are going to be feeling more cautious*, *and if you don’t have a clear policy to go by*, *you might end up saying ‘oh I think you should just have a caesarean section’*.*” (Senior Obs Reg 3)*

*Subtheme 1*.*3 Influence of media—personal and professional consequences*. A perception of Ireland being a litigious country, negative attention from the media and public attitude towards a clinician’s practice with a legal case influenced clinicians’ practice.

“*I think the doctors probably have a little bit more stress on them*. *‘Cos in Ireland at the moment…the media are really out to get maternity services*. *And anything bad that happens*, *whether it’s malpractice or not*, *once something ends up in a coroner’s court the doctors are always named in the media…Especially for the consultants*, *I think that’s a lot of pressure on them*.*” (Mid 3)*

#### Theme 2 Personal preferences versus a threshold—clinician driven factors

Clinicians gave examples of how their personal beliefs, preferences, interpretations of clinical situations, and their practice pattern, influenced their decision-making. These included a clinicians’ level of tolerance and threshold to wait for the natural progression of labour, or intervene early in situations with suspected fetal distress. Three subthemes were identified under the clinician-driven factors; ‘individualised practice versus a judgement call’; ‘consultant obstetrician–a decision-maker versus approver of the decision’; and ‘role of confidence and skills’.

*Subtheme 2*.*1 Individualised practice versus a judgement call*. There were variations in individual clinicians’ interpretation of the overall clinical picture and their threshold to intervene, which influenced the outcome of their decision. Some of these variations were evident among obstetricians’ decision-making for women attending privately and women attending in the public care system.

“*There is a variation in the threshold to intervene* [among obstetricians]. *There are clinicians who are better at thinking from an etiological perspective…That’s the first thing*. *The second thing is there are variations in the tolerance of how long health professionals are prepared to let an abnormal CTG continue…If your threshold is to intervene very quickly…they may make a decision to do a caesarean section…very quickly*.*” (Consultant Obstetrician 1)*

“*They* [consultants in private practice] *just seem to have a lower threshold for section and…my perception is that maybe it’s* [because] *they don’t have to answer to anybody so they’re much quicker just to bailout with the section*.*” (Mid 11)*

Although fetal distress, lack of progress following spontaneous onset of labour or IOL were considered to be the three most common reasons for a first-time CS, clinicians’ personal beliefs in ambiguous situations, for example establishing a diagnosis of labour dystocia or lack of progress following IOL, played a major role in determining the outcome of labour.

“*If* [the woman] *is in the active phase of labour…and…making progress*, *how long they’ve been on syntocinon doesn’t really come into my decision-making*. *If they are in…the latent phase of labour and there is no cervical change…after six hours on maximum syntocinon*, *then I would…consider that to be dystocia*.*” (Consultant Obstetrician 4)*

First-time mothers’ increased age at birth and high BMI were described as being other factors that influenced clinicians’ decision-making.

“*I think our women are very unfit…A lot of…our primigravida aren’t young…healthy and fit and slim*. *They’re…a bit older…a lot heavier…I suppose our diabetes*, *blood pressure all…are on the rise…so therefore our caesareans are on the rise…” (Mid 8)*

“*People have been going through a long*, *hard and expensive process to become pregnant*. *And I think if I had someone in that age group…saying to me I don’t want any risk for this baby…I would be more than happy…to do an elective caesarean section for someone who is 48 and has probably spent 5 or 6 years trying to get to that point to have a healthy baby at term*. *So those are the situations*.*” (Consultant Obstetrician 8)*

*Subtheme 2*.*2 Consultant obstetrician—a decision-maker versus approver of the decision*. Consultant obstetricians’ availability on site was described as a factor that influenced decision-making. In the absence of a consultant on site, the obstetric registrar on call discussed the clinical scenarios with the consultant over the telephone. The final decision on mode of birth was often dependent on the individual obstetric registrar’s interpretation of the clinical scenario and their predetermined view of the possible outcome, and the obstetric consultant’s familiarity with the registrar’s level of expertise.

“*The consultant would be heavily involved* [in the decision-making process] *if they were on site*. *If it’s after hours*, *generally it’s…a discussion over the telephone…If they hear that we’ve done three FBSs [fetal blood samples]…they agree with going for a caesarean section*.*” (Mid 4)*

“*If the consultant is at home and you’re the registrar on the labour ward*, *we all know how you sell the story of the patient*. *You…can tell the same story in two different ways*, *and look for two different outcomes…The consultants*, *even though ultimately it’s their decision*, *they’re relying very heavily that the information that they get from the registrars is correct and appropriate*.*” (Senior Obs Reg 7)*

*Subtheme 2*.*3*. *Role of confidence and skills*. Clinicians’ level of experience and confidence was regarded as a major influence on the overall decision-making process. Balancing between the decisions to proceed with an instrument-assisted vaginal birth versus performing a CS was very much dependent on an obstetrician’s level of confidence and skill, mostly interpreted as a judgement call for a given clinical scenario.

“*If the obstetrician…doesn’t feel confident…they might just say that it’s not suitable for vaginal delivery and then proceed to section*…*or…a midwife manager*, *who feels that an obstetrician doesn’t have the skill…she might suggest that a caesarean would be a better option for the woman*.*” (Mid 7)*

Performing a vaginal breech birth was regarded as a ‘lost skill’ among midwives and obstetricians. When the fetus was in a breech presentation, most midwives and obstetricians stated that an elective, pre-labour CS would be planned.

“*Unfortunately…one big study … has done damage to obstetric practice probably forever*, *the ‘Term Breech Trial’*, *and actually the evidence in that* [study] *isn’t that strong*. *… So*, *I think it is … patient selection that is the problem with breech*. *And I think it is a real shame that all these women are having caesarean section for breech babies*, *and that we are losing our skill in breech delivery because of one study*.*” (Senior Obs Reg 3)*

#### Theme 3 Standardised versus individualised care–a system perspective

Clinicians’ beliefs and their practice within the system and culture of the institution had a major influence on their decision-making. Three subthemes emerged within the system perspective; ‘blending into the system—influence of organisational factors’; ‘Private versus public—a possible difference in practice’; and ‘Women–where do they stand in the process of decision-making?’

*Subtheme 3*.*1 Blending into the system—influence of organisational factors*. Hospital guidelines for IOL, external cephalic version for management of women with breech presentations, etc. were described as a few major factors contributing to the rising rates of CS. Most of the midwives and obstetricians perceived the rates of induction in their institutions to be very high, particularly for first-time mothers and believed that not all IOL were for absolute clinical indications.

“*One of the greatest challenges in modern obstetrics is induction of labour and the significant caesarean section rate in primigravids*, *who have their labour induced*. *So*, *a really important factor… is evaluating…if the induction fails does this woman really warrant a caesarean section*?*” (Consultant Obstetrician 5)*

“*Another direct influence would be the … external cephalic version… guideline that is very restrictive … that’s obviously going to increase the caesarean section rate for breech*.*” (Consultant Obstetrician 5)*

Decision-making for CS was also influenced by the infrastructural limitations, e.g., labour ward capacity and overcrowding and lack of manpower resources with shortage of midwives and obstetricians.

“*it’s the lack of human resources…if you have a very full labour ward and…there are ten women…waiting to come to the labour ward*, *a woman in the emergency room*, *who’s four centimetres and waiting for a bed…If you have a woman who’s been there on the labour ward all day and is making very slow progress*, *for right or wrong you do make decisions based on the other external influences which are…too many patients and…too few staff*.*” (Consultant Obstetrician 5)*

“*The age profile and the skill profile of our midwives would be that they’re quite junior…*. *And they don’t always have the experience to do that* [sound judgement]. *So*, *then they’re reliant upon the clinical midwifery manager*, *who…goes from room to room…it’s very difficult…it* [staffing] *definitely does have an impact*.*” (Mid 5)*

Having an experienced and skilled midwife was described as vital in the process of decision-making. Although decision-making for CS was viewed as a shared process, with obstetricians being the final decision-maker, the midwife being viewed as an advocate varied widely, and depended on the clinical situation and obstetrician on call.

“*It* [the decision-making] *depends on how empowered the woman is and how empowered the midwife is*. *If there is a consultant who believes in the midwife’s role…who is very…supportive of it and includes her in the decision-making you…can achieve a lot there by supporting the woman*, *being on her side*.*” (Mid 13)*

A few obstetricians described decision-making being influenced by midwives’ predetermined view and their tendency to pass over the responsibility to the obstetricians in difficult situations. Conversely, a few others viewed that a senior midwife influenced the decision-making by a junior obstetrician.

“*…sometimes happens*, *where you get a very experienced midwife who puts it to the registrar [obstetric registrar] that this is how they should manage the patient and … really the decision has been driven by the midwife*.*” (Consultant Obstetrician 1*)

*Subtheme 3*.*2*. *Private versus public—a possible difference in practice*. The decision to perform a CS for women attending care under the public category or privately was believed to depend on the individual consultant obstetrician and their practice pattern. However, most consultants regarded their practice as being no different for women attending the private and public healthcare categories. Women booking for private care were, in general, believed to have either a complex medical or obstetrical background which influenced the decision-making on mode of birth.

“*Decision-making process is the same*. *But…the sort of patient that seeks private antenatal care now is different…People*, *particularly for their first pregnancy who choose private care…through reasons of their own*, *do so because they are those older*, [with] *complicated… past history* [and] *medical problem*.*” (Consultant Obstetrician 16)*

There was an inclination to intervene more quickly for women attending the consultant privately compared to women with similar risk profile attending publicly.

“*If you have a consultant obstetrician looking after you*, *they’d be much quicker to bailout of a labour*…*where I think if you have public patients the registrars…have to answer to the consultants and…attend…meetings where…the case might be looked back at*, *they’re more likely to try and prove…that there is fetal distress before…they go to section*.*” (Mid 11)*

Lack of transparency of management and obstetricians’ individualised decisions for women booking under their care, combined with lack of audit of clinical outcomes, were described as being major influencing factors on the high CS rates in women attending obstetricians privately.

“*There’s no one auditing or criticising their [consultants’] practice [of] their private ladies*. *But there is someone criticising their practice on the public ladies…So they tend to…step back a bit more with the public ladies and follow hospital guidelines or national guidelines*.*” (Mid 3)*

“*I think definitely in the private sector there’s probably an easier recourse to a section in a primip rather than in the public sector*.*” (Consultant Obstetrician 8)*

Women under private care were described as having more choice when requesting a CS compared to women booking for public care where CS on request was not an easy option in the absence of medical indications.

“*It* [maternal request] *is definitely higher* [in the private category]*…because I think they get a choice*. *Whereas a public patient…it wouldn’t really be spoken about*.*” (Mid 12)*

“*I would think in a public clinic where you have a woman who states*, *I want a caesarean section for back pain*, *and … you’d strongly encourage her to think of alternatives or to keep an open mind*. *It’s easier to do that when she’s not paying you for her care*.*” (Consultant Obstetrician 1)*

Women in private care were described as having a feeling of being in control of the decision-making.

“*I always think that women who book privately anyway think that they own the consultant and they just make demands and often consultants feel like their hands are tied*.*” (Senior Obs Reg 9)*

‘Convenience’ was perceived as a factor that influenced the decision-making for induction and management of labour and ultimately, a decision to perform a CS, mostly in private care.

“*If they* [women] *think they* [the consultant obstetricians] *are not going to be there…then they’re like*, *‘okay I will go for induction while you are there’ or…‘can we plan my caesarean section while you are there’*. *So*, *I…think private practice is…very different than public*…*” (Mid 4)*

“*I think there is certainly an element of time keeping*, *for private consultants*, *and some of that is unreasonable*. *It’s just at a certain point they want to get home*. *But some of it is reasonable as well*, *in that…they’re expected to be in two places at once*, *as part of their public job…So they just make a decision to [do a CS]” (Senior Obs Reg 7)*

*Subtheme 3*.*3 Women—where do they stand in the process of decision-making*? In general, maternal request for CS was not regarded as being a major factor influencing the decision to perform a CS for first-time mothers. Some obstetricians were open about accepting and approving a woman’s request for a CS when the woman was aware of the associated risks.

“*I completely support maternal request for a caesarean section*, *if they’re aware of the risks associated with doing the procedure*.*” (Consultant Obstetrician 17)*

Midwives sometimes were unsure if all first-time mothers were provided with all the information prior to consenting to a CS and regarded professionals’ role as being vital and crucial in explaining to a woman about CS to obtain informed consent prior to the procedure.

“*I mean it’s their* [women’s] *choice*, *it’s their body*…*But I think we as professionals have a duty to make them fully aware of what is involved and the long-term consequence of having a caesarean*, *that it is a major operation*, *that things can go wrong*.*” (Mid 2)*

With regards to women’s active involvement in the decision-making process a few clinicians perceived that women were more informed, and hence they played an active role in the decision-making process. Others disagreed and described women’s role as being passive and driven by the information presented to them by the clinical team.

“*Women probably…aren’t as involved in the…actual making of the decision*. *It’s…discussed with them as…the plan of care and this is what’s going to happen*. *It’s only women who are very adamant or very strong…might have a very strong birth plan*, *or birth preferences*, *who are very well informed that might push for…longer time” (Mid 7)*

“*So*, *it’s hard for them* [women] *because they…don’t feel empowered to actually make that decision*. *They’re pretty much presented with our version of the story…their involvement is quite limited*.*” (Mid 13)*

Women’s predetermined view of CS as a safe, easy option and their underlying fear of labour or childbirth had an influence on their decision-making for CS.

“*There is a perception by women that it [CS] is an easy option*. *I don’t think they look at the long-term health consequences*, *they’re not aware that the fertility reduces after your first caesarean section*, *scar tissue*, *pain down the line…They think it’s the easy option*.*” (Mid 2)*

“*I think caesarean section they [women] see as this clean*, *neat*, *tidy thing to happen*. *So…some women…do think…it is the better option”*. *(Senior Obs Reg 3)*

Advancements in anaesthesia, and a perceived low morbidity and mortality associated with planned CSs had an influence on the belief system among clinicians and women, and this influenced clinicians’ acceptance of women’s request as a reasonable choice, particularly with individual profiles of older women with history of infertility.

“*It drives me insane when they keep comparing us to the Netherlands…I mean…they are…taller than the average Irish person…healthier [and]…slimmer*. *We are fat*, *old and short*. *That’s basically the Irish population of women who are giving birth*. *So it is a huge influence in terms of what*, *what the caesarean section rate should be for your country…*.*” (Consultant Obstetrician 10)*

“*it’s very important that you tease out the particular indicators for it and…living in the western world as we do with you know*, *with low morbidity and mortality related to elective caesarean section and related to advances in anaesthesia*, *there may be*…*many patients*…*maternal request caesarean section is an entirely legitimate choice for them” (Consultant Obstetrician 5)*

## Discussion

Clinicians’ beliefs and attitudes towards CS were key drivers in the decision-making process for CS consistent with other studies [16, 21, 22]. Decision-making for CS was influenced by clinicians’ perceived fear of adverse outcomes from vaginal birth and subsequent litigation, their individual interpretation of clinical situations, practice pattern, convenience, women’s type of care (private or public) and organisational guidelines, for example, criteria for inducing labour, diagnosis and management of fetal distress and labour dystocia.

In recent years, a number of possible influencing factors in the decision-making process to perform a CS have been considered. Often there is ambiguity around what health professionals believe are clinical indications for CS [16]. Changing risk profiles and maternal characteristics, such as increasing maternal age and high BMI [23–26], treatment for infertility [27], are reported as contributing to the rise in CS, which resonate with clinicians’ views in this study. Change in maternal demographics partly contributes to the decision-making for CS; however, this does not fully explain the overall decision-making, and rising CS rates in nulliparous women [25]. Despite the changing demographics of childbearing women a few countries have maintained an acceptable rate of CS, for example, in Sweden, the CS rates have stayed between 15–18% for decades [28], despite an increase in average maternal age [29] and obesity [30]. Challenging the current practices has been suggested as a way to promote normality and optimise normal birth among women with obesity [31].

High rates of CS for women with fetal breech presentation and the practices around management of breech were described as factors in the decision to perform a CS. Clinicians viewed performing vaginal breech birth as a *‘lost skill’* and attributed this to the publication of findings of the ‘Term Breech Trial’ [32], despite several critiques [33, 34], which had *‘damaged’* the practice around management of women with breech presentations forever. A few studies added to the controversies reporting an increased risk of neonatal morbidities following a vaginal breech birth compared to planned CS for women with breech presentations [35, 36]. Despite guidelines from the Health Service Executive (HSE) [37] in Ireland and the National Institute of Healthcare and Excellence (NICE) [38] to conduct vaginal breech births and perform external cephalic version (ECV) for all women with singleton and uncomplicated pregnancy, the practice has remained unchanged in Ireland. Clinicians viewed it as challenging to reverse the current trend in practice due to their lack of necessary skills to facilitate vaginal breech births.

Many clinical factors, taken together within an organisational context or system of practice had an influence on the decision-making process for CS. IOL, for example, was perceived to be a major contributing factor. High rates of IOL with flexible criteria and ambiguity in reasons for inducing labour, leading to more CSs because of lack of progress following IOL, were viewed as influencing the overall rise of CS rates. Despite women’s negative birth experiences associated with lack of progress following IOL [39], clinicians in the study attributed high rates of IOL to pressure from women, and a belief among women about IOL being the right way to end a pregnancy, and clinicians’ own beliefs.

Although disputed by a few consultant obstetricians, most clinicians reported an obvious difference in public versus private system of care, with more individualised care for women in private practice, that resonate with findings from previous studies [16, 24, 40, 41]. Increased risk of interventions has been identified to be a significant factor for high rates of CS among women choosing private care [42]. Clinicians attributed this difference to the lack of auditing of private practice and outcomes for women in private care, consistent with other studies [43, 44]. Not fully accounted for medical/obstetrical risks [25, 45–49], obstetricians’ decision-making in private care, in this study, was influenced to a large extent by *‘convenience’* [16].

The presence of a consultant obstetrician on the labour ward out of hours was viewed as an important factor in the decision-making process consistent with other literature [16, 50, 51]. Consultants in the study described themselves as *‘being at the mercy’* of what was communicated to them by the obstetric registrar on call, and their approval of the decision was mostly influenced by the level of experience of the obstetric registrar on call and the time of day/night. Clinicians regarded communication as being a key factor in the process of decision-making. Whether it was communication between a junior and senior obstetrician, or an obstetrician and a midwife, or clinicians and women, it had a substantial influence in the decision-making process. At clinician level, communicating a clinical scenario to a consultant over the phone for final decision-making or for a second opinion varied from one obstetrician to another. Although ‘*shared decision-making*’ in maternity care is gaining emphasis in the recent times, there are barriers in ‘*communication*’ and the ‘*discourse*’ in the process of decision-making [15]. Clinicians’ communication with women and the way information is presented play a vital role in the process of decision-making, and women’s involvement in the process [52]. Use of a specialised language in ‘*institutional discourse’* is described as a form of abstraction that removes one from the reality behind the words [53]. For example, common phrases in clinical practice such as, ‘*failure to progress*’, ‘*fetal distress*’, ‘*cephalopelvic disproportion*’, ‘*lack of growth*’, *‘failed induction of labour’* overlook what is ‘*real*’, and drive women to ‘*agree*’ to and ‘*go with the flow*’ of professionals’ recommendations [52]. With regards to CS on maternal request, while a few midwives in the study believed that all clinicians had a professional duty to ensure that women were aware of the consequences of having a CS, a few obstetricians viewed CS by maternal request as a ‘legitimate choice’ especially with advances in anaesthesia and low morbidity and mortality related to elective CS. In general, maternal request was not perceived as a major factor in the decision to perform a CS for a first-time mother; however, most clinicians in the study said they would agree to perform a CS on request when the woman was aware of the risks involved. There are disparities with regards to maternal requests for CS as a factor contributing to the rising CS rates [52] with emphasis on the inadequate acknowledgement of obstetric factors in relation to women’s requests or preferences.

There was consensus around clinicians’ perceived fear of adverse outcomes and/or subsequent litigation as being the key driver in the decision-making process consistent with other literature [16]. Although a few clinicians did not view ‘*fear*’ as an influential factor in their own practice, a few midwives described ‘*fear*’ as a *‘learned behaviour’* from senior colleagues or the environment, and many attributed it to a previous ‘*bad experience*’ or *‘media portrayal’* of incidents in Ireland’s maternity service leading to a *‘defensive and safe practice’*. The concept of safety will need to be debated against decision-making to justify the care for women and practice being ‘*evidence-based*’ versus ‘*fear-based*’, and related to clinicians’ preference, attitude, practice pattern and convenience, largely supported by other studies [51, 54–56]. Ireland is ranked 22 out of 179 countries as being a safe place to give birth [57], yet questions have been raised in the media in the past in relation to shortcomings in maternity services for mothers giving birth in Ireland [58]. Clinicians, often not being in a position to explain their individual clinical circumstances, described increasing concerns about the power of social media and its negative impact on their short-term and long-term practice that resonate with other literature [16]. Obstetric care providers’ preferences and attitudes, and a belief that CS is safer than vaginal births despite ongoing debate surrounding inappropriate CSs, are key factors influencing their preference and decision to perform CSs [49, 59]. There were variations in practice among clinicians. For example, while some attempted to manage signs of fetal distress conservatively with a change of position or intravenous fluid infusion, others preferred to intervene “too much, too soon” [14] through repeated vaginal examinations and fetal blood sampling at an early stage, which eventually determined the outcome of their decision.

Although limited to Irish clinicians and their views of factors influencing decision-making for first-time mothers, findings resonate with what has been reported in a large systematic review for all women across other countries [16]. The study was conducted in three sites and, whilst the findings are applicable to these sites, they may resonate with other sites and with hospitals within Ireland. However, because of the unique structure and organisation of care within the maternity care system in Ireland, the findings may have limited applicability to other countries. There is limited research on Irish clinicians’ views of what factors influence their decision to perform a CS, therefore, the strength of this study is the unique presentation of insight into the complexities associated with the decision to perform a CS for first-time mothers from multiple perspective of the key stakeholders and decision-makers (midwives and obstetricians).

## Conclusion

Clinicians’ personal beliefs, attitudes towards CS, perceived fear, interpretation and practice pattern were a few key drivers in the decision-making process for CS for first-time mothers. The decision-making was further influenced by the culture of practice within an organisation, hospital guidelines, and a possible difference in practice for women across different types of care. Findings will help clinicians reflect on their day-to-day practice through identification of potentially modifiable factors that influence their decision-making for CS for first-time mothers, including a reduction of “too much, too soon” types of care. This has a potential to help women understand the multitude of factors that can lead to a decision to perform a CS. The complex nature of decision-making will enable maternity care providers, policymakers and researchers consider broader issues related to organisational, socio-cultural and political context when seeking solutions to stop, if not reverse, the rising CS rates. There is potential to implement changes in practice through devising future intervention studies and development of the ‘*next step action*’ to reduce any inappropriate and/or unnecessary CSs for first-time mothers, and repeat CSs in subsequent pregnancies.

## Supporting information

S1 Appendix Study information for participants. (DOC)

S2 Appendix Willingness to participate form and consent form. (DOCX)

S3 Appendix Interview guide. (DOCX)

S4 Appendix Sample codes and descriptions. (DOCX)

S1 Table Location and current role of participants. (DOCX)

S2 Table Themes and sub-themes. (DOCX)
