# Supplementary material for: Clinicians’ views of factors influencing decision-making for CS for first-time mothers—A qualitative descriptive study
Source: PLoS One. 2022 Dec 28;17(12):e0279403. doi: 10.1371/journal.pone.0279403 (PMC9797090; doi:10.1371/journal.pone.0279403)
Supplement: S1 Appendix — (DOC) [file pone.0279403.s001.doc]

**Information Booklet**

Your invitation to take part in a study on

**Caesarean section in nulliparous women: Factors influencing the decision-making process and outcomes for women - the MAMMI Study Caesarean Section Strand.**

The study has been approved by the Research Ethics Committees of Trinity College Dublin, Rotunda Hospital, Galway University Hospital and Coombe Women and Infants University Hospital.

MAMMI stands for **M**aternal health **A**nd **M**aternal **M**orbidity in **I**reland.

If you have any questions about this study, please contact researcher Sunita Panda at xxxxxxxxxx.

**Contents**

[What is the MAMMI study? 4](#__RefHeading___Toc454531466)

[What sort of questions will you ask me? 4](#__RefHeading___Toc454531468)

[Who else is taking part in this study? 4](#__RefHeading___Toc454531469)

[What does taking part in the study mean for me? 4](#__RefHeading___Toc454531470)

[Are there any risks for me? 5](#__RefHeading___Toc454531471)

[Are there any benefits for me? 5](#__RefHeading___Toc454531472)

[Can anyone take part in the study? 5](#__RefHeading___Toc454531473)

[How will you protect my personal information? 5](#__RefHeading___Toc454531474)

[What do I do next? 5](#__RefHeading___Toc454531476)

[Can I leave the study? 6](#__RefHeading___Toc454531477)

[How can I get in touch with you? 6](#__RefHeading___Toc454531478)

**WHY HAVE I BEEN GIVEN THIS BOOKLET**

You were given this booklet because you are employed in the Rotunda Hospital, Galway University Hospital or the Coombe Women and Infants University Hospital (CWIUH), and have experience of caring for women giving birth by caesarean section. This booklet tells you about the MAMMI study, and the Caesarean Section (CS) strand, and what it means if you decide to take part in one-to-one interviews about the factors influencing the decision-making process for caesarean section in nulliparous women.

# What is the MAMMI study?

MAMMI stands for **M**aternal health **A**nd **M**aternal **M**orbidity in **I**reland. It is a study looking into the health and health problems of first-time mothers during pregnancy and during the first year after the birth.

The caesarean section strand of the study is asking midwives, obstetricians and women to take part in one-to-one interviews about what they think are the factors that influence the decision to perform a caesarean section (CS) in first-time mothers.

# Why are you doing this study?

By doing this study, I want to find out:

what health problems, if any, women who birth by caesarean section experience during the 12 months postpartum;

what health and other services, if any, women use when they have health problems;

how to improve women’s health during and after pregnancy and birth by caesarean section;

how women and clinicians make decisions about giving birth by caesarean section; and

what factors influence the decision for first-time mothers to birth by caesarean section (CS).

# What sort of questions will you ask me?

The number of first-time mothers giving birth by caesarean section has increased in the last decade. Now, almost one third of first-time mothers in Ireland give birth by caesarean section, and I would like to ask you what you think are the factors and circumstances that influence the decision to perform a caesarean section in these women.

# Who else is taking part in this study?

I am inviting 2600 women, aged 18 years and over, who are having their first baby to take part in the MAMMI study. A small number of these women (about 20-25) who have birthed by CS will be asked to take part in one-to-one interviews about their experiences, their health and the factors they think influenced the decision to perform the caesarean section.

# What does taking part in the study mean for me?

If you decide to take part in the one-to-one interview of this study, I will ask you to:

text/call me, or complete the ‘willingness to participate’ form that came with this booklet;

take part in an audio recorded one-to-one interview which will take approximately 30-45 minutes of your time.

# Are there any risks for me?

I do not foresee any risks with taking part in this study. However, if a problem arises during the interview (for example, information comes to light that a woman or baby has been harmed) I must tell the appropriate people in the relevant study site.

# Are there any benefits for me?

The study will not benefit you personally. The information you give will be pooled with the information given by all other clinicians and women in the study and will help us to understand the factors that influence the decision-making for caesarean section for first-time mothers, which may help improve care for mothers and babies in the future.

# Can anyone take part in the study?

To take part in the study you must be employed in the Rotunda Hospital, the CWIUH or the Galway University Hospital, and be willing to be take part in a one-to-one interview.

Midwives must be working on the labour ward at the time of the study, and obstetricians must be responsible for making decisions about performing caesarean sections in the hospital.

# How will you protect my personal information?

I will keep all the information you give me private and confidential. I will audio record the one-to-one interview and, when I make a paper copy of the recording, I will replace your name with a unique study number (a code). I will offer you the chance to see this paper copy so that you can confirm it is an accurate copy of the interview. I will then transfer the recording to a secure hard drive and then delete the information from the recording device once analysis of data has been completed. I will use your study number at all times to identify you and I will not use any information that might identify you personally in any publications arising from the study. I will store the paper copy of the interview in a locked cabinet, in a locked office in an area to which few people have access.

I will use passwords and anti-virus software to protect the information held on the computer.

All members of the study team who have access to this information must sign a confidentiality agreement form. I will only disclose your personal details in **exceptional circumstances** for example, if information emerges that a mother or baby is being harmed, or if you complain about the researcher.

# What happens to the information at the end of the study?

I will publish the findings from the study and may give talks about the findings at healthcare conferences. It will not be possible to identify you or your answers in these publications or talks.

# What do I do next?

If you are interested in taking part in a one-to-one interview, please text/call me or complete the ‘**Willingness to participate’** form that came with this booklet and post it to me in the FREEPOST envelope provided.

I will then contact you to answer any questions you have, arrange a suitable time and place for interview and gain your written consent.

# Can I leave the study?

Taking part in the study is voluntary. You can withdraw from the study at any time without giving a reason.

# How can I get in touch with you?

My name is Sunita Panda and you can contact me on xxxxxxxxxx by calling or texting.

You can also get information on our website www.mammi.ie.
