# Supplementary material for: Clinicians’ views of factors influencing decision-making for CS for first-time mothers—A qualitative descriptive study
Source: PLoS One. 2022 Dec 28;17(12):e0279403. doi: 10.1371/journal.pone.0279403 (PMC9797090; doi:10.1371/journal.pone.0279403)
Supplement: S2 Appendix — (DOCX) [file pone.0279403.s002.docx]

**Willingness to Participate Form**

**Research title:**

**Caesarean section in nulliparous women: Factors influencing the decision-making process and outcomes for women - the MAMMI Study Caesarean Section Strand.**

**Researcher: Sunita Panda Tel: xxxxxxxxxx**

**DECLARATION by participant**

I have read the information booklet for this research study and I understand the contents.

I understand that completing this form indicates my willingness to be contacted by the researcher, Sunita Panda and, that when I am contacted, I will be given an opportunity to ask questions about the study.

I understand that I can change my mind and decide not to take part at any stage.

**Clinician’s NAME** ……………………………………………………………………………........................

**Contact Address**……………………………………………………….........................

**Phone number:**…….…………………………...................**Email**:...............................................

**Participant’s signature**: ……………………………………**Date:**..............................

**CONSENT FORM**

**Research title:**

**Caesarean section in nulliparous women: Factors influencing the decision-making process and outcomes for women - the MAMMI Study Caesarean Section Strand.**

**Researcher: Sunita Panda Tel: xxxxxxxxxx**

| DECLARATION by participant: Please tick ( X o r √) and provide your initials | | |
| --- | --- | --- |
| 1. | I have read the information booklet for this research study and I understand the contents. | Yes [ ] No [ ] initials [ ] |
| 2. | I have had the opportunity to ask questions and all my questions have been answered to my satisfaction. | Yes [ ] No [ ] initials [ ] |
| 3. | I fully understand that my participation is completely voluntary and that I am free to withdraw from the study at any time (prior to publication). | Yes [ ] No [ ] initials [ ] |
| 4. | I understand that I am being asked to participate in an interview on the factors that influence the decision to perform a caesarean section in first-time mothers and I consent to this. | Yes [ ] No [ ] initials [ ] |
| 5. | I understand that I will be given an opportunity to review the transcript of such an interview(s) to confirm accuracy. | Yes [ ] No [ ] initials [ ] |
| 6. | I understand that the transcript will not identify me by name but will use the study code and that the original digital recording will be erased once the accuracy of the transcript has been confirmed. | Yes [ ] No [ ] initials [ ] |
| 7. | I understand that information from this research will be published but that I will not be identified as a participant in this research in any publication. | Yes [ ] No [ ] initials [ ] |
| 8. | I agree that information obtained from me in this research which has been coded so as not to identify me may be stored and used for the purpose of future research which will have obtained Research Ethics Committee approval without the need for further consent from myself. | Yes [ ] No [ ] initials [ ] |

| 9. | I understand that my personal details (name and address and other identifying information that links my identity to the study data) will be destroyed when this study is complete. | Yes [ | ] No [ | ] initials [ | ] |
| --- | --- | --- | --- | --- | --- |
| 10. | I understand that the researchers undertaking this research will hold in confidence and securely all collected data and other relevant information. | Yes [ | ] No [ | ] initials [ | ] |
| 11. | I freely and voluntarily consent to participating in this research study. | Yes [ | ] No [ | ] initials [ | ] |

**PARTICIPANT'S NAME** ……………………………………………………………..

**Contact Address**……………………………………………………………………………………

**Phone number:**…….……………………………………… **Email:**…………………..………………..

**Participant’s signature**:…………………………....... **Date:**........................................

Name of person taking consent: ………………... Signature: ……................. Date:……….......................

Researcher: ………………….…............................ Signature: …………………….. Date:…….……..................

One copy of this form must be retained by the participant and one copy must be retained by the researcher
