# Supplementary material for: Clinicians’ views of factors influencing decision-making for CS for first-time mothers—A qualitative descriptive study
Source: PLoS One. 2022 Dec 28;17(12):e0279403. doi: 10.1371/journal.pone.0279403 (PMC9797090; doi:10.1371/journal.pone.0279403)
Supplement: S3 Appendix — (DOCX) [file pone.0279403.s003.docx]

| **Interview guide for clinicians** | | **Contemporaneous notes and post interview notes** |
| --- | --- | --- |
| 1. **Decision made by obstetric registrar on-call/senior obstetrician on call.**   **1.a.Seeking second opinion**  **1.b.Discussed with obstetric consultant**  **1.c.Women’s view taken into account, or asked for** | How do you classify Caesarean section? |  |
|  | What do you think are the most common reasons for CS in nulliparous women |  |
|  | What is the decision-making process for CS? |  |
|  | Who makes the decision for a CS in the institution you are employed in? |  |
|  | | |
| **Foetal distress**  **Failure to progress**  **Organisational factors (hospital policy and protocols)**  **Staffing issues**  **Lack of skill among clinicians**  **Lack of resources**  **Social issues (fear of litigation)**  **Maternal request**  **Time of the day or night (shift issues)**  **Health care coverage (insurance status of women)** | What is your role in the decision-making for a CS in nulliparous women? |  |
|  | Are there any other factors which may have an influence in decision-making for a CS in nulliparous women? |  |
|  | How do you feel about the way decision to perform CS are made?  Anything else you would like to say? |  |
