# Supplementary material for: Clinicians’ views of factors influencing decision-making for CS for first-time mothers—A qualitative descriptive study
Source: PLoS One. 2022 Dec 28;17(12):e0279403. doi: 10.1371/journal.pone.0279403 (PMC9797090; doi:10.1371/journal.pone.0279403)
Supplement: S4 Appendix — (DOCX) [file pone.0279403.s004.docx]

**Clinicians’ views of factors influencing decision-making for CS – Codes and description using NVivo**

| **Codes** | **Description** |
| --- | --- |
| Defensive practice as a result of fear of adverse outcomes and litigation   - Uncertainties associated with vaginal birth - Protect self - Protecting the women - Learned behaviour from senior colleagues | This code described clinicians’ fear in relation to adverse outcomes associated with uncertainties from vaginal births and subsequent litigation. This was attributed to lack of support when things go wrong resulting in a more defensive practice for self-protection as well as for protecting the women from any unexpected adverse outcomes. This also described fear as a learned behaviour from other colleagues and clinical environment. This ultimately influenced clinicians’ decision-making. |
| Past experience and its influence   - Own experience of dealing with adverse outcomes in past - Witnessed colleagues’ bad experience of going through bad outcomes and legal issues | A clinician’s personal experience of dealing with an adverse outcome or subsequent litigation, as well as knowing someone who had to go through a bad outcome and resulting litigation influenced his/her practice forever. |
| Cultural and societal influence   - Social media and its influence - Maternity service being discussed in Irish media - A blaming culture | This code included descriptions about how media and a ‘blaming’ culture of practice in Ireland influenced clinicians’ decision-making. |
| Clinicians’ experience   - Junior versus senior - Role of experience | This category includes codes relating to difference in decision-making based on obstetricians’ experience. |
| Skills and confidence   - Midwives’ experience and confidence - Obstetricians’ experience and confidence | Midwives’ experience and confidence in supporting a woman in labour and an obstetricians’ skill in managing a difficult vaginal birth in second stage of labour was described in this category. |
| Preference and practice pattern   - Personal preference of obstetricians and midwives - Obstetricians’ practice pattern | Clinicians’ own way of managing a situation based on their preference and practice pattern was described in this category. |
| Who makes the final decision – the consultant obstetrician or the senior registrar on call?   - Obstetricians relying on the information received - On-call obstetricians’ interpretation of the situation when the consultant is not in the hospital | Obstetricians described the consultant’s decision mostly based on the communication between the consultant and senior registrar over phone when the consultant was not present in the hospital. This category also included the discussion around importance of the presence of consultant obstetrician in the hospital round the clock. |
| Low threshold to intervene   - Clinicians less tolerant of any deviation from normal - Clinicians’ personal interpretation - Low threshold to intervene early | Some obstetricians had a low threshold to intervene early, for example in case of early signs of fetal distress, doing an FBS to check fetal oxygenation status, while others were good at managing conservatively through changing the women’s position, close observation, and allowing the women to progress in labour, etc. |
| Perception of the reason to perform a CS   - Maternal demographics such as age, BMI and treatment for infertility - Management of breech presentation | Increased maternal age, high BMI, treatment for infertility were some reasons perceived by most clinicians as clinical reasons to induce labour and subsequent CS, when the IOL failed. |
| Induction of labour   - High rates of IOL in Irish maternity units - Failed IOL – major reason for CS - Flexible criteria to induce labour | This category includes codes related to IOL being one of the major reasons for unnecessary and avoidable CSs. Most clinicians raised concerns related to the reasons for inducing labour and the existing flexibility in criteria for IOL. |
| Difference in practice for women in public versus private care. Obstetricians’ convenience   - Lack of auditing of private practice - Women with more choices in private care - Obstetric consultants’ convenience – a major factor | This category included codes related to private consultants doing their own practice for women in their care, women with more choices in private care, obstetric consultants’ convenience, etc. |
| Institutional disclosure   - Hospital infrastructural facilities - Getting blended into the system and culture of practice within the organisation - Hospital policies and guidelines | This was a broad category which included limitations related to hospital guidelines and management policy within a system perspective of clinicians’ blending into the system of practice. |
| Women’s role is crucial in decision-making process   - Different views on women’s role - Maternal request – not a major factor | This described the codes related to different views from clinicians on women’s role. Some viewed women’s roles as vital and other believed women having a limited role in the process of decision-making. Maternal request was not viewed as a major factor. |
