# Supplementary material for: Clinicians’ views of factors influencing decision-making for CS for first-time mothers—A qualitative descriptive study
Source: PLoS One. 2022 Dec 28;17(12):e0279403. doi: 10.1371/journal.pone.0279403 (PMC9797090; doi:10.1371/journal.pone.0279403)
Supplement: S1 Table — (DOCX) [file pone.0279403.s005.docx]

S1 Table Location and current role of participants

| **Participant** | **Current role** | **Number of participants** | | | **Total years of experience in current role** | | |
| --- | --- | --- | --- | --- | --- | --- | --- |
|  |  | **Site 1** | **Site 2** | **Site 3** | **Less than 5 years** | **5 to 10 years** | **> 10 years** |
| Obstetrician | Consultant Obstetrician | 4 | 3 | 4 | 3 | 5 | 3 |
|  | Senior Obstetric Registrar | 2 | 3 | 4 | 4 | 5 | - |
| Midwife | Clinical Midwife Manager | 3 | 1 | 3 | - | 3 | 4 |
|  | Staff Midwife | 2 | 3 | 3 | 3 | 3 | 2 |
