# Supplementary material for: Clinicians’ views of factors influencing decision-making for CS for first-time mothers—A qualitative descriptive study
Source: PLoS One. 2022 Dec 28;17(12):e0279403. doi: 10.1371/journal.pone.0279403 (PMC9797090; doi:10.1371/journal.pone.0279403)
Supplement: S2 Table — (DOCX) [file pone.0279403.s006.docx]

S2 Table Themes and sub-themes

| **Themes** | **Subthemes** |
| --- | --- |
| 1. **A fear factor** | 1.1. Fear of litigation and/or adverse outcome  1.2. Ever present - influence of past experience  1.3. Influence of media - personal and professional consequences |
| **2. Personal preferences versus a threshold - clinician driven factors** | 2.1. A variation in interpretation and practice pattern  2.2 Consultant obstetrician - a decision-maker versus approver of the decision  2.3. Role of confidence and skills |
| **3. Standardised versus individualised care – a system perspective** | 3.1. Blending into the system - Influence of organisational factors  3.2. Private versus public - a possible difference in practice  3.3. Women - where do they stand in the process of decision-making? |
